# Leaf Stable Isotope and Nutrient Status of Temperate Mangroves As Ecological Indicators to Assess Anthropogenic Activity and Recovery from Eutrophication

**DOI:** 10.3389/fpls.2016.01922

**Published:** 2016-12-23

**Authors:** Iana Gritcan, Mark Duxbury, Sebastian Leuzinger, Andrea C. Alfaro

**Affiliations:** Institute for Applied Ecology New Zealand, School of Science, Faculty of Health and Environmental Sciences, Auckland University of TechnologyAuckland, New Zealand

**Keywords:** environmental assessment, marine pollution, leaf nitrogen content, leaf phosphorus content, nitrogen stable isotope ratio, herbarium isotope samples

## Abstract

We measured nitrogen stable isotope values (δ^15^N), and total phosphorus (%P) and total nitrogen (%N) contents in leaves of the temperate mangrove (*Avicennia marina* sp. *australasica*) from three coastal ecosystems exposed to various levels of human impact (Manukau, high; Mangawhai, low; and Waitemata, intermediate) in northern New Zealand. We measured δ^15^N values around 10‰ in environments where the major terrestrial water inputs are sewage. The highest average total nitrogen contents and δ^15^N values were found in the Auckland city region (Manukau Harbour) at 2.2%N and 9.9‰, respectively. The lowest values were found in Mangawhai Harbour, situated about 80 km north of Auckland city, at 2.0%N and 5.2‰, respectively. In the Waitemata Harbour, also located in Auckland city but with less exposure to human derived sewage inputs, both parameters were intermediate, at 2.1%N and 6.4‰. Total phosphorus contents did not vary significantly. Additionally, analysis of historical mangrove leaf herbarium samples obtained from the Auckland War Memorial Museum indicated that a reduction in both leaf total nitrogen and δ^15^N content has occurred over the past 100 years in Auckland’s harbors. Collectively, these results suggest that anthropogenically derived nitrogen has had a significant impact on mangrove nutrient status in Auckland harbors over the last 100 years. The observed decrease in nitrogenous nutrients probably occurred due to sewage system improvements. We suggest that mangrove plant physiological response to nutrient excess could be used as an indicator of long-term eutrophication trends. Monitoring leaf nutrient status in mangroves can be used to assess environmental stress (sewage, eutrophication) on coastal ecosystems heavily impacted by human activities. Moreover, nitrogen and phosphorus leaf contents can be used to assess levels of available nutrients in the surrounding environments.

## Introduction

Human activities continue to affect coastal ecosystems throughout the world at alarming rates. Habitat destruction (e.g., deforestation, urbanization) and eutrophication (e.g., agricultural runoff, sewage inputs) have been identified as major factors that affect water quality in aquatic ecosystems within coastal areas (Heaton, 1986; Valiela et al., 1992, 1997; Nixon et al., 1996; McClelland and Valiela, 1998; Verhoeven et al., 2006). Indeed, excessive nutrient inputs (eutrophication) from intensive agricultural activity and growing populations within coastal regions usually result in rapid degradation of water quality and modifications of ecological features (Schindler, 2006; McGlathery et al., 2007; Heisler et al., 2008; Smith and Schindler, 2009).

Ecological consequences of eutrophication are directly associated with nutrient contents (e.g., nitrogen, phosphorus) in the tissues of organisms present in these ecosystems. Previous studies have shown that plant tissue nutrient composition is linked to the nutrient availability in the surrounding environment (Aerts and Chapin, 2000; Güsewell, 2004), making the nutrient status of those organisms susceptible to any nutrient excess. Aquatic coastal and estuarine primary producers (e.g., phytoplankton, macroalgae, and seagrasses) serve as ‘coastal filters’ and are able to absorb and sequester nutrients into plant biomass (Costanzo et al., 2001; Nixon et al., 2001; McGlathery et al., 2007).

Mangrove stands could play an important role in mitigating eutrophication in coastal settings as they have been described as nutrient limited ecosystems with a generally positive physiological response to nutrient addition (Alongi, 2009; Reef et al., 2010). For example, previous laboratory (Boto et al., 1985; Naidoo, 1987; Yates et al., 2002; Alongi, 2011) and field studies (Boto and Wellington, 1983; Feller, 1995; Lovelock et al., 2007b; Naidoo, 2009) have shown that mangrove leaf nutrient status (nitrogen and phosphorus contents) correlate well with levels of nutrient addition and/or natural variability in nutrient concentrations within the environment. Mangrove plants, which can grow extensively and form stable stands within most protected coastal regions of the tropics and subtropics could be of particular importance for remediation.

To study the ability of organisms in taking up nutrients derived from anthropogenic sources, nitrogen stable isotope values (δ^15^N) can be used as they provide reliable information about the source (anthropogenic vs. natural) of nutrients within coastal areas (Lindau et al., 1989; McClelland and Valiela, 1998; Fry et al., 2000; Costanzo et al., 2001; Thimdee et al., 2002; Rogers, 2003; Fry, 2006). δ^15^N values of around +10‰ are attributable to human and animal sewage, which is generally correlated with the presence of excess nutrients (eutrophication; Fry et al., 2000; Costanzo et al., 2001). Elevated δ^15^N values arise due to the presence of excess nutrients in the environment allowing increased isotope fractionation via increased volatilisation of ammonia and/or increased microbial processing. Indeed, Rogers (2003) used nitrogen stable isotope values within mussel and limpet samples to show how fast the aquatic system recovered from sewage outfall closures. Also, Costanzo et al. (2001) showed that different dominant plant groups (e.g., seagrasses, macroalgae, and mangroves) could be used as anthropogenic activity indicators (sewage input vs. pristine environments) within a habitat.

The mangrove genus *Avicennia* is widely distributed across the tropical and sub-tropical regions, including Australia, the Arabian Peninsula, Brazil, China, India, Indonesia, Japan, Malaysia, the Phillipines, Pakistan, the southern United States and Central America, South Africa, and New Zealand (Duke et al., 1998). *Avicennia*’s characteristic occurrence in the intertidal ecotone makes it an ideal indicator genus for monitoring eutrophication in coastal habitats in large areas of the world. Indeed, population growth in coastal areas where *Avicennia* is present, makes the implementation of eutrophication monitoring methods increasingly important, since population growth is generally linked to increased environmental eutrophication (Ansari and Gill, 2014). The methods described in the current research are easily applied and widely applicable to environmental monitoring in the tropical and subtropical coastal regions, where a large proportion of the world’s population resides.

It is well documented that nutrient concentrations in New Zealand coastal waters have increased greatly in the past 100 years due to various anthropogenic activities (Cooper and Thomsen, 1983; Vant, 1997; Hart et al., 2004; Heggie and Savage, 2009; Thrush et al., 2013), and some areas around the highly populated Auckland region have been described as having ‘poor’ water quality (Walker and Vaughan, 2014). However, the effect of such changes on the nutrient status of the endemic mangrove *Avicennia marina* sp. *australasica* is understudied, especially with regard to the role of nutrient inputs from sewage, agriculture practices, and livestock production. Additionally, mangrove stands have been expanding within most northern New Zealand estuaries (Green et al., 2003; Schwarz, 2003; Stokes et al., 2010) as the result of increasing catchment-derived sediment inputs (Lovelock et al., 2007a; Morrisey et al., 2010), with undocumented effects on the nutrient status of these coastal ecosystems. In addition to contemporary sampling from northern New Zealand estuaries, Auckland Museum (AM) specimen collections are also available to investigate long-term variations in mangrove nutrient status using herbarium samples of the past 100 years.

Thus, the aim of this research is to quantify foliar nutrient parameters (total nitrogen, total phosphorus, and nitrogen stable isotope ratio values) in temperate mangroves of northern New Zealand, and relate these measurements to sources and magnitudes of human activity (e.g., sewage input, agricultural runoff). A further aim is to investigate the potential use of mangrove plants as indicators of eutrophication in long term monitoring of aquatic ecosystems.

## Materials and Methods

### Study Sites

This study was conducted in northern New Zealand. The city of Auckland is situated on an isthmus between two inlets, the Waitemata Harbour to the north-east and the Manukau Harbour to the south-west. Even though these two inlets are only about 1.6 km apart at their closest point on the isthmus, there is no direct connection between the two, with the Waitemata opening to the Hauraki Gulf and the Manukau to the Tasman Sea. The third main site sampled was Mangawhai harbour located 81 km north of Auckland. The sites were selected because they are in close proximity to anthropogenic activity hot spots (e.g., water treatment plant) and they covered a wide demographic and geographic region. Also, in the present study, an attempt was made to locate mangroves growing in relatively pristine conditions, by obtaining samples from nature reserves on Great Barrier and Motu Kaikoura Islands, located approximately 90 km offshore from central Auckland.

### Contemporary Leaf Collections

Initially, mangrove leaf samples were collected in 2013 from 18 sites throughout Manukau Harbour, Auckland and nine sites in the Mangawhai Harbour Estuary, Mangawhai. In 2014, leaves were collected from 12 sites in Waitemata Harbour, Auckland. A larger collection was made in 2015, with 30 sites throughout Manukau Harbour, 29 sites in Waitemata Harbour, 10 sites in Mangawhai Harbour and five sites in the Great Barrier Island archipelago. In all years, collections were made during the winter season (April–September) so that the leaves would be mature following the peak summer growing season. Sampling points were recorded with geographic coordinates and are listed in **Supplementary Table 1**. A total of 10 leaves per tree were sampled from 10 trees spaced approximately 10 m apart at each sampling site. For consistency, the leaves were selected to be fully mature, but not senescent. All leaves were oven dried at 65°C for 3 days.

### Auckland Museum Herbarium Samples

To compare contemporary and past leaf nutrients levels, historical herbarium mangrove leaves were obtained from the AM specimen collections and compared to contemporary samples from the same locations. When no samples from the same locations could be obtained or exact information about the herbarium sample location was missing, the closest geographical location was sampled or the mean value of the entire site was used. These leaves were mainly from Waitemata Harbour (18 samples), Manukau Harbour (3 samples), and one sample from Great Barrier Island (**Supplementary Table 2**). The collection dates for the herbarium material ranged from 1863 to 1990. The leaves were analyzed for nitrogen content and nitrogen stable isotope ratio. Total phosphorus contents were not measured in herbarium mangrove leaves, since the amount of leaf sample available from the museum was limited.

### Total Nitrogen, Total Phosphorous, and Stable Isotope Analyses

Dried composite leaf samples were ground to a fine powder with a ball mill (PM 100, Retsch, © Retsch GmbH) and sifted through a 200 μm pore size sieve. Analysis for total phosphorus (%P) was conducted using wet digestion in concentrated HNO_3_ (McQuaker et al., 1979), followed by quantification using an inductively coupled plasma atomic emission spectrometer (ICP-AES, Varian Liberty AX Series II, USA). A National Institute of Standards and Technology (NIST) Peach Leaf standard reference material (SRM1547) was used as a quality control in all analytical batches. A total of 0.1–0.5 grams (g) of each composite leaf sample was sent to the Waikato University Stable Isotope Unit for total nitrogen (%N) and stable isotope (δ^15^N) analyses.

Herbarium samples from the AM were brought to the laboratory and ground to a fine powder in the presence of liquid nitrogen. These samples were analyzed using the same procedures as mentioned above. Only one leaf per herbarium voucher could be analyzed, which could have caused some additional variation.

### Statistical Analyses

Leaf %N, %P, N/P ratio, and δ^15^N values are reported as mean ± standard error (SE). To test for differences between polluted and less polluted harbors using contemporary leaves, ANOVA was used to compare %N, %P, N/P ratio, and δ^15^N values between the three harbor locations in the main trial year (2015). Residuals of ANOVA were normal and homogeneous so that no transformations were considered necessary. ANOVAs were followed by Tukey’s HSD tests when significant differences were detected. To test for changes in leaf nutrients over time by comparing contemporary and historical samples, %N, and δ^15^N of Herbarium and inter-annual variability samples were tested for normality and *t*-tests and Wilcoxon rank-sum tests were used accordingly. Inter-annual variability was assessed using *t*-test, since all variables were normal. For all statistical analyses and plotting, the R software version 3.2.1 was used (R Core Team, 2015).

## Results

**FIGURE 1:**
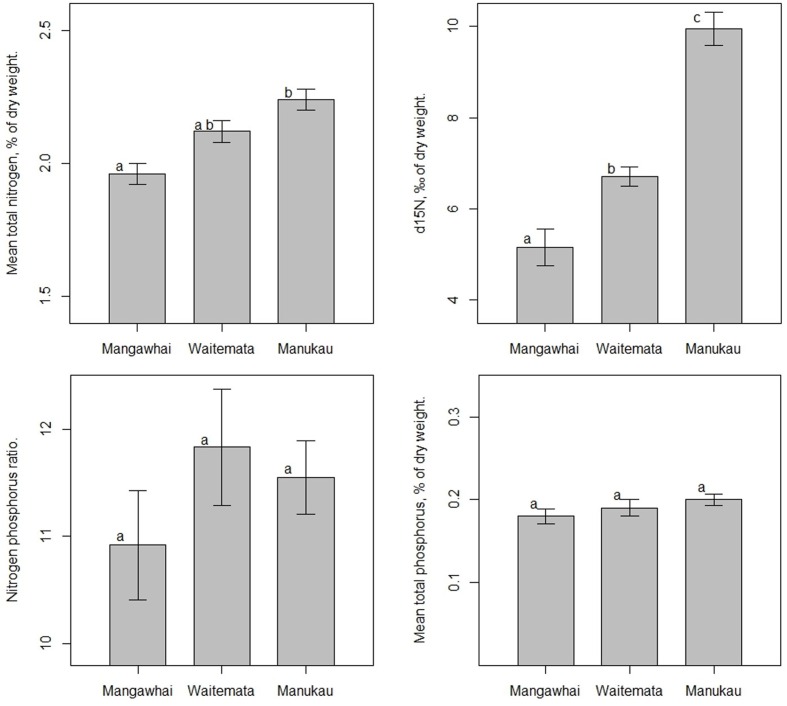
**Mean (±SE) nutrient parameters (Mean total nitrogen, δ^15^N, Nitrogen phosphorus ratio, Mean total phosphorus) in mangrove (A*vicennia marina* sp. *australasica*) leaves within three harbors (Mangawhai *n* = 10, Waitemata *n* = 29, Manukau *n* = 30) during the main sample collection in winter 2015.** ANOVAs were followed by Tukey’s HSD tests when significant differences were detected. Values with different letters are significantly different at *p* < 0.01.

**FIGURE 2:**
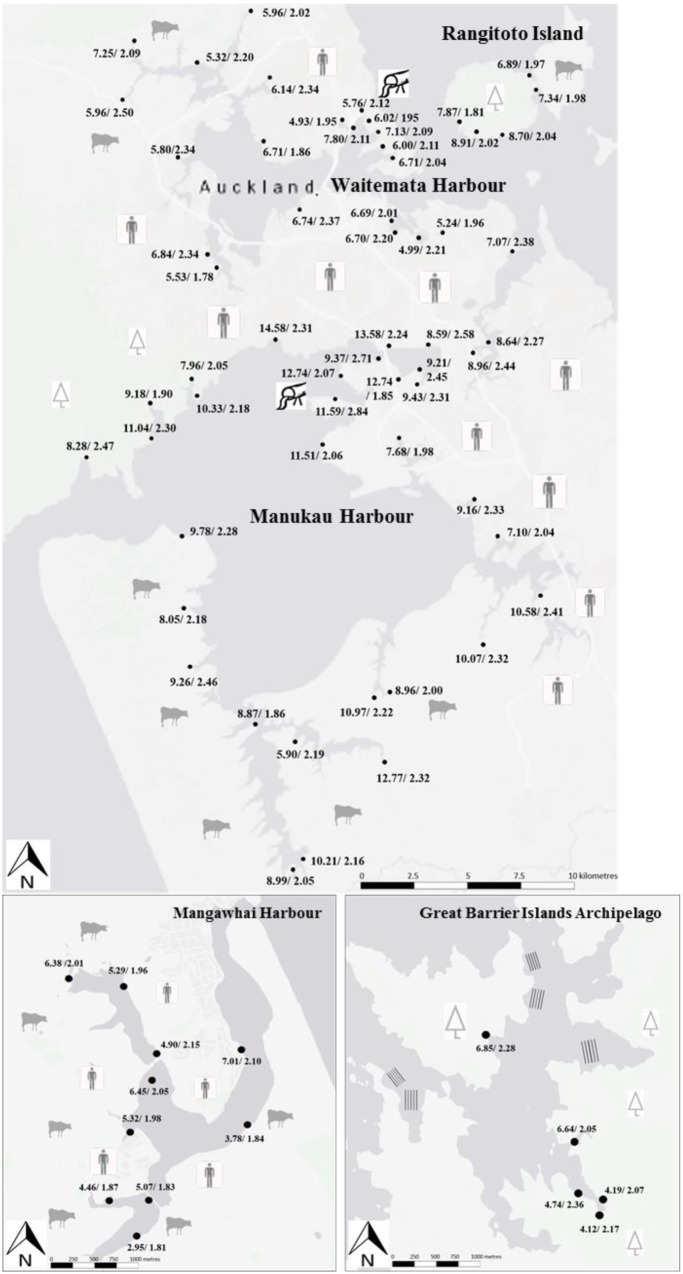
**Nitrogen stable isotope ratios (‰)/total nitrogen (%N), of dry weights in mangrove (*A. marina* sp. *australasica*) leaves at individual sampling sites around Auckland city (top map), Mangawhai Harbour and Great Barrier Island Archipelago during the main sample collections in winter 2015**.

**FIGURE 3:**
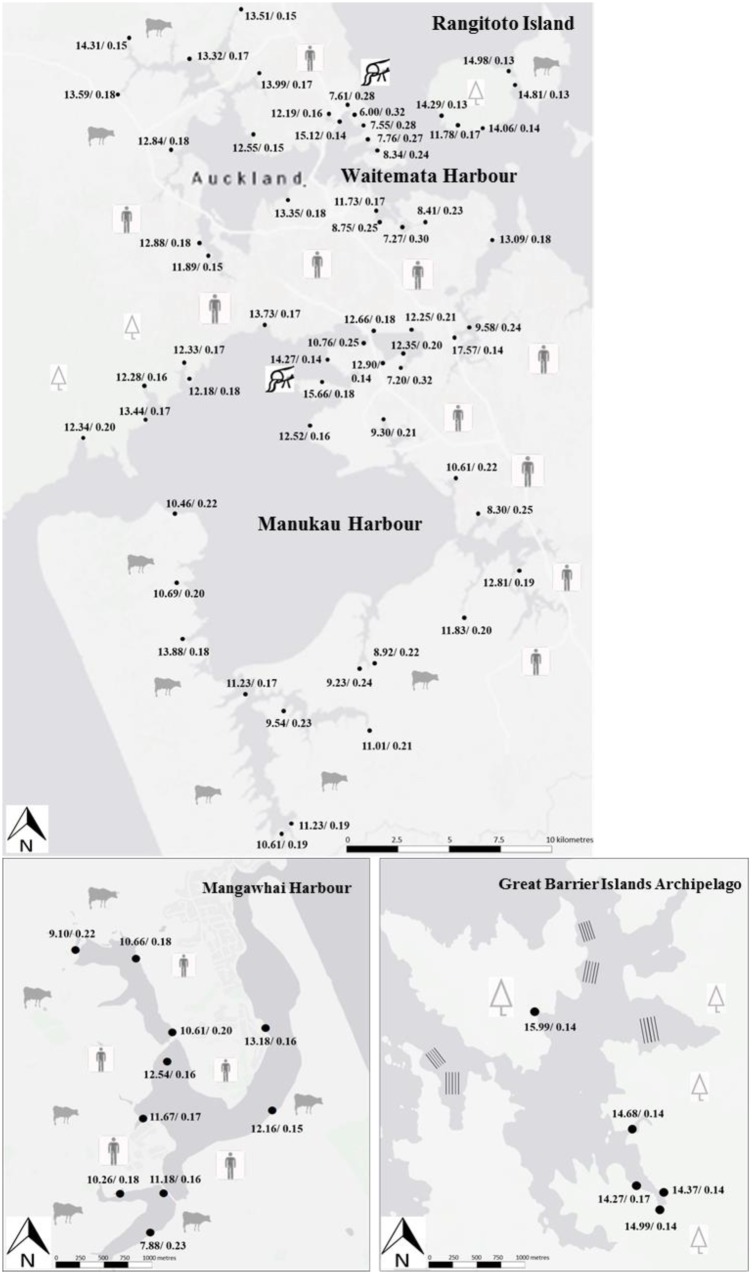
**Nitrogen phosphorus ratio/total phosphorus (%P), of dry weights in mangrove (*A. marina* sp. *australasica*) leaves at individual sampling sites around Auckland city (top map), Mangawhai Harbour and Great Barrier Island Archipelago during the main sample collections in winter 2015**.

For the main sampling year (2015), the highest leaf total nitrogen contents and nitrogen stable isotope (δ^15^N) values were found at Manukau (2.2 ± 0.1%N, 9.9 ± 0.4‰, respectively) and the lowest at Mangawhai (2.0 ± 0.1%N, 5.2 ± 0.4‰, respectively; **Figures 1** and **2**). The differences in both %N and δ^15^N ratios were highly significant (*p* < 0.01, Tukey’s HSD test). In addition, the lowest δ^15^N value observed at Manukau (7.1‰) was higher than all the δ^15^N values at Mangawhai, i.e., there was no overlap in δ^15^N values between the two harbors. The %N contents and δ^15^N values for the Waitemata mangrove leaves (**Figures 1** and **2**) were intermediate in value between those for Manukau and Mangawhai (2.1 ± 0.1%%N and 6.4 ± 0.2‰). In contrast to the nitrogen values, the leaf phosphorus contents were not significantly different among the three sites, with 0.18 ± 0.01% at Mangawhai, 0.19 ± 0.01% at Waitemata and 0.20 ± 0.01% at Manukau (**Figures 1** and **3**). Leaf nitrogen-phosphorus ratios also did not show any significant trend among sites (**Figures 1** and **3**).

The %N contents and δ^15^N ratios at Great Barrier Island were 2.2 ± 0.1%, and 5.3 ± 0.6‰, respectively. Great Barrier Island mangroves had the lowest total phosphorus contents in their leaves (0.15 ± 0.01%) and the highest nitrogen to phosphorus ratios (14.9 ± 0.3) of any sampled site (**Figures 2** and **3**).

### Inter-Annual Variability

When comparing parameters measured during the preliminary study years (2013, 2014) with the data from the same locations gathered in the main sampling year (2015), measurements did not differ greatly, except for the nitrogen stable isotope (δ^15^N) ratios between Manukau samples (*p*-value < 0.05, *t*-test or Wilcoxon test, **Table 1**).

**Table 1:** Comparison of data from the preliminary and main sampling collections.

Sites	Preliminary sampling (2013 and 2014)		Main sampling (2015)	
		
	TN, % dry mass	δ^15^N, ‰ dry mass	TN, % dry mass	δ^15^N, ‰ dry mass
Manukau (2013, *n* = 18)	2.4 ± 0.1	10.4 ± 0.5	2.2 ± 0.1	9.8 ± 0.4
Waitemata (2014, *n* = 12)	2.2 ± 0.1	6.3 ± 0.2	2.1 ± 0.1	6.5 ± 0.3^∗^
Mangawhai (2013, *n* = 9)	2.2 ± 0.1	5.3 ± 0.3	2.0 ± 0.1	5.4 ± 0.3


*^∗^*p*-value < 0.05, *t*-test or Wilcoxon test.*

### Museum Samples

The highest nitrogen stable isotope value in the historical museum samples was found in the Manukau Harbour at Ihumatao Street point (18.2‰) in the mid-1980s. The highest total nitrogen content was found in Purewa Bush point (3.1%) in the mid-1950s.

**FIGURE 4:**
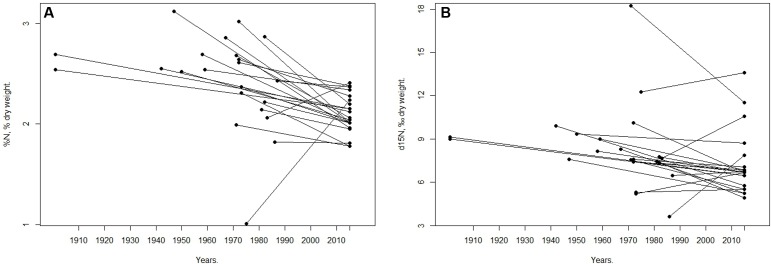
**General trend between nitrogen stable isotope ratios, ‰** **(A)** and total nitrogen, % **(B)** in historical Auckland Museum (AM) herbarium samples and samples from the present study conducted in winter 2015. In both diagrams, the lines link historical herbarium samples collected at various dates to leaf samples from the same site in 2015.

In general, nutrient parameters of mangrove leaves found in the present study were lower than historical values, except for two sampling points at the Manukau site (**Figure 4**). The mean total nitrogen content of herbarium samples was significantly higher at 2.6 ± 0.1%, compared to 2.1 ± 0.1% in the 2015 samples (*p*-value < 0.001, *t*-test). The stable nitrogen isotope ratio in the herbarium samples were also significantly higher (7.9 ± 0.4‰) in comparison with values found in the 2015 samples (6.2 ± 0.2‰) (*p*-value = 0.001, Wilcoxon test).

## Discussion

### Leaf δ^15^N Values at the Three Contemporary Harbor Sites

In general for a variety of plants growing under pristine natural conditions in various areas of the world, the δ^15^N of tree leaves ranges from -8 to +3‰ (Peterson and Fry, 1987) and leaf values lying within this range have been observed in *A. marina* mangroves (1.6‰, 2.2‰) growing on an off-shore island in Australia (Costanzo et al., 2001). In contrast, in the present study, the three studied sites in northern New Zealand had mean δ^15^N values ranging from +5 to +10‰, and no single site had a δ^15^N mean value within the natural range.

Two potential sources of the nitrogen which may have caused these enhanced δ^15^N values are agricultural practices and human sewage. With respect to agriculture, New Zealand has the third highest use of nitrogenous fertilizer in the OECD countries after South Korea and Japan at 27 kg/ha (OECD, 2015). Excess deposition of urea on land from either fertilization or by livestock urination results in an increased probability of kinetic isotope fractionation either via ammonia volatilisation or by microbial denitrification due to system “leakiness” associated with the excess nitrogen (Heaton, 1986; Fry et al., 2003). Thus, both in New Zealand and internationally, high levels of dairy and animal farming are associated with high levels of δ^15^N in nitrate produced by microbial processes (Komor and Anderson, 1993; Harrington et al., 1998). Plants, in turn, absorb this ^15^N enriched nitrate, leading to elevated δ^15^N values in plant tissues. For example in a study of δ^15^N and % N in *Rhizophora* mangrove leaves in Florida, the highest δ^15^N and %N values were observed where canals draining agricultural lands deliver high-nitrate waters to fringing mangrove marshes. Mangroves growing adjacent to agricultural canals had δ^15^N values in the range +11 to +16‰, whereas mangroves growing at a more pristine site had values that ranged from -5 to +2‰ (Fry et al., 2000). A similar elevation in δ^15^N values has been associated with proximity to human sewage sources in both aquatic plants in general (McClelland and Valiela, 1998) and *A. marina* mangrove stands in particular (Costanzo et al., 2001). While direct measurements of the ^15^N signature of water running into the harbors is technically feasible there would be a high intra-day and inter-day variability in such data due to changes in temporal events such as rainfall and land use. The advantage of using mangroves as ecological indicators is that the mangrove acts as a continuous sampler, integrating, and storing ^15^N over the lifetime of the leaf. In addition mangroves leaves are more easily accessible than other potential indicators (such as macroalgae) and the trees themselves have long lives, potentially allowing sampling from the same plant over several decades.

Based on the δ^15^N values observed at the three sites, it appears that Mangawhai at 5.2‰ is receiving mildly elevated inputs of anthropogenic nitrogen at +2‰ above the upper natural background level of +3‰ (Peterson and Fry, 1987), while the Manukau at +9.9 ‰ is receiving strong anthropogenic inputs at almost +7‰ above the natural background level. The Waitemata Harbour lies between these two values at +6.4‰. The main source of anthropogenic nitrogen at Mangawhai is likely to be from farm animals since Mangawhai has a relatively small human population of 1,329 (Census 2013). In contrast, the city of Auckland, where the Waitemata and Manukau harbours are located, is New Zealand’s largest city with a population of 1.4 million within the wider Auckland region (Census 2013). The highly significant difference in δ^15^N values between the two Auckland sites (6.4 vs. 9.9‰) is, therefore, a potential indicator that the Manukau Harbour receives greater anthropogenic nitrogen inputs.

The most likely source for this difference is the main Auckland sewage treatment plant which is located adjacent to the Manukau Harbour and discharges an effluent output of c. 120,000,000 m^3^ of treated sewage per year into the harbor (with an average total nitrogen concentration of 8.3 gm^-3^ in winter and 6.8 gm^-3^ in summer; Watercare Service Limited, 2013). Also, the Manukau Harbour has a higher proportion of adjacent farmland compared to the Waitemata Harbour.

### Total Leaf Nitrogen and Phosphorus Values at the Three Contemporary harbor Sites

The Waitemata and Manukau Harbours have elevated leaf total nitrogen contents compared to Mangawhai Harbour, which supports the δ^15^N observations that indicate higher anthropogenic nitrogen inputs at these two sites compared to Mangawhai. The mangrove leaf N data from the present study also correlate with Auckland water quality monitoring data. According to the 2013 Auckland Council Marine Water Quality Annual Report, calculated total inorganic nitrogen concentrations of Manukau Harbour surface water were significantly higher than in the Waitemata Harbour (0.15 and 0.05 mgL^-1^, respectively; Walker and Vaughan, 2014).

The majority of studies in natural ecosystems have found that mangrove productivity is primarily limited by nitrogen and occasionally by phosphorus (Reef et al., 2010). A value for the level of nitrogen and phosphorus at which growth limitation of *A. marina* occurs has not been determined in natural ecosystems since many variables may impact growth (Reef et al., 2010; Alongi, 2011). However, Alongi (2011) studied the effect of nitrogen and phosphorus on the growth of *A. marina* and five other mangrove species under controlled tidal hydroponic conditions. Plants were grown in seawater with a range of nitrogen concentrations, and the growth rates and leaf nutrient contents were determined. The leaf nitrogen contents of *A. marina* increased with increasing concentrations of nitrogen in the seawater solutions, and ranged from a low of 1.1% at low nitrogen supplementation rates to a high of 3.4% total leaf nitrogen at very high supply rates. *A. marina* displayed an S-shaped nitrogen dependent growth curve, which plateaued at a nitrogen supply rate of 10 mmol m^-2^d^-1^, which resulted in a measured average leaf content of 2.1% nitrogen.

Thus, under otherwise optimal conditions, the growth of *A. marina* may be nitrogen limited up to a leaf content of slightly less than 2.1% N. In the present study it is noteworthy that the average *A. marina* leaf nitrogen contents from the two Auckland sites exceed this value, implying that the natural ecological nitrogen limitation on growth would be removed from these mangroves (under optimum salinity conditions). Alongi (2011) commented that the nitrogen solution concentration that gave this supply rate was likely to be the maximum that would be obtained under natural environmental conditions, but might be exceeded in polluted ecosystems, which gives further credence to the δ^15^N observations that indicate anthropogenic nitrogen inputs into the mangroves of the two sites close to Auckland.

### Leaf N/P Ratios at the Three Contemporary Harbor Sites

Generally, nitrogen or phosphorus limitation in plants has been reported as an N/P ratio of less than 10 or greater than 20, respectively (Güsewell, 2004). Furthermore, an absolute value of less than 0.1% phosphorus in leaves is also generally indicative of phosphorus limitation (Güsewell, 2004). Based on these criteria none of the mangrove sites in the three contemporary harbor sites surveyed in the current study showed clear phosphorus limitation. However, while the mean N/P values for the three sites indicate that nitrogen limitation is unlikely to be present overall, several plots at all three sites may be nitrogen limited since 17 of the 69 plots surveyed had N/P ratios of less than 10.

### Great Barrier Island Mangroves

None of the mangroves in the 69 locations sampled in the three main harbor sites had δ^15^N values in the -8 to +3‰ range, which is expected under pristine natural conditions (Fry, 2006). Costanzo et al. (2001) observed high δ^15^N values in mangrove leaves associated with sewage outfalls, but also observed low values (1.6–2.2‰) on an offshore island located approximately 20 km away from the main site. In contrast, in the present study the leaf δ^15^N and %N values from the off shore Great Barrier Island mangroves sites were relatively high, with means of 5.3‰ and 2.2%. Paradoxically, however, the %P values were relatively low with a mean of 0.15% P. This resulted in high N/P ratios, with a mean of 14.9. Indeed, this 14.9 N/P ratio approaches the values of 15–18 seen in old growth New Zealand pristine forests, which are typically phosphorus limited (Richardson et al., 2004; Parfitt et al., 2005). Thus, the phosphorus limitation results at this site suggest that the area might be pristine, while conversely the nitrogen results suggest the opposite.

### Herbarium Leaf Analyses

General trends between herbarium samples and contemporary collections strongly suggest that there has been a decline in nitrogen stable isotope ratios and total nitrogen content in mangrove leaves over the past 100 years in Auckland’s estuaries. This finding is consistent with herbarium studies that have observed a decline in leaf %N and δ^15^N in a variety of plant species in other parts of the world over the last century (Peñuelas and Matamala, 1990; Peñuelas and Estiarte, 1997; Peñuelas and Filella, 2001; McLauchlan et al., 2010). The authors in these herbarium studies ascribed the decline in leaf %N and δ^15^N to either increased absorption of elevated atmospheric CO_2_ related to anthropogenic activities over the last century, or to increased absorption of anthropogenically derived atmospheric nitrogen species depleted in ^15^N. However, in the present study, the decline is more likely a result of improvements in the Auckland sewage treatment system over the past 100 years (Fitzmaurice, 2009), potentially leading to reduced inputs of δ^15^N and %N.

In contrast to the present study, other herbarium studies used plant samples largely obtained from relatively pristine ecosystems where nitrogen was likely limiting. Under these conditions, elevated CO_2_ levels have been shown to cause reductions in plant leaf nitrogen content (McGuire et al., 1995). However, under elevated CO_2_ conditions and ample nitrogen availability, both reductions and increases in leaf and plant %N have been observed (McGuire et al., 1995). Likewise, in the herbarium study of Peñuelas and Filella (2001), low (-10 to 0‰) leaf δ^15^N was observed in samples from relatively pristine areas in the Mediterranean. In that study, precipitation derived anthropogenic nitrogen species depleted in ^15^N were likely to make a large contribution against a low natural background. Atmospheric nitrogen supply has been calculated to represent a high proportion (36–53%) of the N incorporated into biomass in Mediterranean systems due to low soil moisture, in contrast to more temperate ecosystems where nitrogen species, such as ammonium or nitrate, are derived from groundwater (Peñuelas and Filella, 2001). Therefore, in the present study, where consistently high δ^15^N values were observed, the major source of nitrogen is likely to be ammonium or nitrate from microbially processed urea in water derived from sewage or farm runoff, which is typically enriched in δ^15^N (rather than depleted in ^15^N as is atmospheric ammonium or nitrate). The observed historic decline in leaf δ^15^N and %N is hence more likely to be related to a reduction in sewage impact rather than to atmospherically derived nitrogen or to elevated CO_2_ levels. However, impacts of elevated CO_2_ levels over the last century cannot be excluded and the observed decline in leaf %N and δ^15^N may well be due to a combination of both elevated CO_2_ levels and improvements in the Auckland sewage system.

Pitt et al. (2009) found that mangrove plants, unlike algae and crabs, had no detectable response in δ^15^N values to an improvement in sewage outfall quality in the Moreton Bay catchment, Australia, up to 2 years after an upgrade had been completed. They attributed the lack of change to the slower growth rate and turnover time of nitrogen in mangroves compared to algae. Mangroves source most of their N via their roots from sediments that can accumulate large quantities of N. In addition, mangroves are likely to store N for an extended period and unlike algae, mangroves recycle a proportion of their N internally since they resorb up to 64% of N from senescing leaves prior to abscission. Here, we see no or minimal changes occurring over short time periods (1–2 years), but significant changes occurring over longer time periods (decades) as indicated by the herbarium samples. Thus, algae may be more suitable for monitoring short term trends in eutrophication (Pitt et al., 2009) whereas mangroves are long lived and integrate nitrogen signatures across large spatiotemporal scales, making them more suitable for monitoring long term trends.

## Conclusion

Mangroves are able to absorb nutrients in coastal waters, which is reflected in their tissue nutrient status. Anthropogenically derived discharges into coastal waters, such as animal and human sewage, and agricultural runoff could provide additional nutrients to mangrove plants, which may in turn affect growth, since under natural conditions mangrove productivity is often nitrogen limited. In the present study, we observed contrasts in both leaf nitrogen contents and stable isotope ratios in *Avicennia* mangroves which correspond with human activities such as sewage discharge and farming on both spatial and temporal scales. The *Avicennia* mangrove genus is widely distributed across the tropical and sub-tropical regions of the world. Our finding that *A. marina* leaves can provide a robust medium-term record of changes in anthropogenic N discharge, indicates that it is a useful indicator species for monitoring of eutrophication in coastal habitats where population growth is likely to exacerbate eutrophication. Implementation of eutrophication monitoring methods such as those employed in the present study will become increasingly important in monitoring of anthropogenic impacts on ecological systems internationally.

## Author Contributions

Substantial contributions to the conception or design of the work; or the acquisition, analysis, or interpretation of data for the work (IG, MD, SL, and AA); Drafting the work or revising it critically for important intellectual content (IG, MD, SL, and AA); Final approval of the version to be published (IG, MD, SL, and AA); Agreement to be accountable for all aspects of the work in ensuring that questions related to the accuracy or integrity of any part of the work are appropriately investigated and resolved (IG, MD, SL, and AA).

## Conflict of Interest Statement

The authors declare that the research was conducted in the absence of any commercial or financial relationships that could be construed as a potential conflict of interest.
