# Supplementary material for: Leaf Stable Isotope and Nutrient Status of Temperate Mangroves As Ecological Indicators to Assess Anthropogenic Activity and Recovery from Eutrophication
Source: Front Plant Sci. 2016 Dec 23;7:1922. doi: 10.3389/fpls.2016.01922 (PMC5179504; doi:10.3389/fpls.2016.01922)
Supplement: Supplementary file 1 [file Table_1.docx]

**Supplementary tables**

**Table 1: Nutrient parameters at individual sampling locations during the main mangrove nutrient trial from April-October 2015 in Waitemata, Manukau, and Mangawhai harbours in northern New Zealand.**

| **Date collected** | **Street location** | **Marine location** | **Coordinates** | **TP, % dry weight** | **TN, % dry weight** | | **δ^15^N, ‰** |
| --- | --- | --- | --- | --- | --- | --- | --- |
| **Waitemata Harbour** | | | | | | | |
| 7/05/2015 | St Peters Street | Tuff Crater (Dwarf) | -36.801377, 174.751402 | 0.16 | 4.93 | | 1.95 |
| 7/05/2015 | Exmouth Road | Tuff Crater (Tall) | -36.804405, 174.759107 | 0.14 | 7.8 | | 2.11 |
| 7/05/2015 | Balmain Road | Soldiers Bay, Birkenhead | -36.813342, 174.698193 | 0.15 | 6.71 | | 1.86 |
| 7/05/2015 | Manuka Rd | Oruamo Creek, Glenfield | -36.777850, 174.698435 | 0.17 | 6.14 | | 2.34 |
| 7/05/2015 | Wharf Rd | Lucas Creek, Albany | -36.732961, 174.687831 | 0.15 | 5.96 | | 2.02 |
| 7/05/2015 | Chatham Avenue | Chatham Reserve, Poremoremo | -36.765793, 174.648290 | 0.17 | 5.32 | | 2.20 |
| 7/05/2015 | Wharf Rd | Rangitopuni Stream, Riverhead | -36.756720, 174.598452 | 0.15 | 7.25 | | 2.09 |
| 7/05/2015 | Dale Road | Brigham Creek, Whenuapai | -36.786027, 174.598837 | 0.18 | 5.96 | | 2.50 |
| 14/05/2015 | Parawai Crescent | Coxs Creek | -36.850657, 174.727947 | 0.18 | 6.74 | | 2.37 |
| 14/05/2015 | Glen Marine Parade | Whau River, Glendene (Dwarf) | -36.881097, 174.660162 | 0.15 | 5.53 | | 1.78 |
| 14/05/2015 | Glen Marine Parade | Whau River, Glendene (Tall) | -36.880886, 174.658507 | 0.18 | 6.84 | | 2.34 |
| 14/05/2015 | Moire Rd | Henderson Creek,Henderson | -36.824154, 174.635069 | 0.18 | 5.80 | | 2.34 |
| 14/05/2015 | Shore Road | Hobson Bay ,Remuera | -36.863276, 174.789393 | 0.17 | 6.69 | | 2.01 |
| 14/05/2015 | West Tamaki Road | Tahuna Torea Reserve, Wai o Taiki Bay, Glendowie | -36.872494, 174.885754 | 0.18 | 7.07 | | 2.38 |
| 14/05/2015 | Princes Street (East) | Seaside Park, Otahuhu | -36.931732, 174.865515 | 0.14 | 8.96 | | 2.44 |
| 16/08/2015 | Greydene Place, Takapuna | Auburn Reserve walkway | -36.791201, 174.766965 | 0.28 | 5.76 | | 2.12 |
| 16/08/2015 | Harley Close, Takapuna | Creek | -36.797126, 174.772594 | 0.32 | 6.02 | | 1.95 |
| 16/08/2015 | Francis street, Takapuna | Shoal Bay | -36.803183, 174.780547 | 0.28 | 7.13 | | 2.09 |
| 16/08/2015 | Kawerau Avenue, Devonport | Ngataringa Bay | -36.815459, 174.785910 | 0.27 | 6.00 | | 2.11 |
| 16/08/2015 | Ngataringa Park, Devonport | Lake Road Bridge | -36.817955, 174.794182 | 0.24 | 6.71 | | 2.04 |
| 7/09/2015 | Church street, Otahuhu | Tamaki Estuary | -36.935997, 174.845149 | 0.24 | 8.64 | | 2.27 |
| 26/09/2015 | Waitaramoa Reserve | Hobson Bay ,Remuera | -36.865751, 174.794834 | 0.25 | 6.70 | | 2.20 |
| 26/09/2015 | Palmers in Remuera, walkway | Hobson Bay ,Remuera | -36.865044, 174.807779 | 0.30 | 4.99 | | 2.21 |
| 26/09/2015 | Kepa Bush Reserve, Purewa | Purewa | -36.863954, 174.827796 | 0.23 | 5.24 | | 1.96 |
| 27/09/2015 | Islington Bay | Rangitoto Island | -36.775703, 174.897284 | 0.13 | 7.34 | | 1.98 |
| 27/09/2015 | Rangitoto Warf | Rangitoto Island | -36.806625, 174.862581 | 0.14 | 8.70 | | 2.04 |
| 27/09/2015 | Mangrove Bridge | Rangitoto Island | -36.805938, 174.851080 | 0.17 | 8.91 | | 2.02 |
| 27/09/2015 | Coast Guard Bay | Rangitoto Island | -36.790776, 174.831076 | 0.13 | 7.87 | | 1.81 |
| 27/09/2015 | Gardiner Gap | Rangitoto Island | -36.770939, 174.893522 | 0.13 | 6.89 | | 1.97 |
| **Manukau Harbour** | |  |  | | | | |
| 14/05/2015 | Norana Avenue | Harania Creek, Mangere | -36.945102, 174.816248 | 0.20 | 9.21 | | 2.45 |
| 14/05/2015 | Norana Avenue | Mangere-Seashore | -36.943872, 174.811313 | 0.14 | 12.74 | | 1.85 |
| 14/05/2015 | Hugo Johnstone Drive | Pikes Point-Onehunga | -36.929091, 174.820272 | 0.21 | 8.59 | | 2.58 |
| 9/06/2015 | Arapito Road | Little Muddy Creek, Laingholm | -36.959014, 174.646605 | 0.18 | 10.33 | | 2.18 |
| 9/06/2015 | Landing Road | Little Muddy Creek, Laingholm | -36.950275, 174.646272 | 0.17 | 7.96 | | 2.05 |
| 9/06/2015 | Huia Road | Big Muddy Creek (Nihotapu Dam) | -36.966978, 174.615878 | 0.16 | 9.18 | | 1.90 |
| 9/06/2015 | Armour Road | Big Muddy Creek (Armour Bay) | -36.973610, 174.619152 | 0.17 | 11.04 | | 2.30 |
| 9/06/2015 | Huia Dam Road | Huia Stream | -36.997863, 174.566588 | 0.20 | 8.28 | | 2.47 |
| 9/06/2105 | Ambury Road | Ambury Farm Park Seashore | -36.948857, 174.756389 | 0.14 | 12.74 | | 2.07 |
| 9/06/2015 | Island Road | Puketutu Island, Mangere | -36.961788, 174.755108 | 0.18 | 11.59 | | 2.84 |
| 9/07/2015 | Linwood Road, Glasson Bridge | Whangamaire Stream, Karaka | -37.101615, 174.864016 | 0.20 | 10.07 | | 2.32 |
| 9/07/2015 | Capriole Crescent | Clarks Creek (Eastern arm), Kingseat | -37.126285, 174.788238 | 0.22 | 8.96 | | 2.00 |
| 9/07/2015 | McKenzie Road | Clarks Creek (Western arm),Kingseat | -37.135613, 174.782305 | 0.24 | 10.97 | | 2.22 |
| 9/07/2015 | Racecourse Road | Waiuku River (Eastern Waiuku Estuary) | -37.235014, 174.730393 | 0.19 | 10.21 | | 2.16 |
| 9/07/2015 | Rangiwhea Road | Waiuku River (Western Waiuku Estuary) | -37.234623, 174.728397 | 0.19 | 8.99 | | 2.05 |
| 9/07/2015 | Featon Avenue | Awhitu Park Creek, Awhitu | -37.086042, 174.646441 | 0.20 | 8.05 | | 2.18 |
| 9/07/2015 | Poaka Road | Matakawau Creek (Bay) | -37.116289, 174.656715 | 0.18 | 9.26 | | 2.46 |
| 9/07/2015 | Big Bay Road | Big Bay Creek | -37.044112, 174.638830 | 0.22 | 9.78 | | 2.28 |
| 10/07/2015 | Lewis Street | Blockhouse Bay | -36.926531, 174.706971 | 0.17 | 14.58 | | 2.31 |
| 10/07/2015 | Alfred Street | Mangere Inlet, Onehunga | -36.931112, 174.795638 | 0.18 | 13.58 | | 2.24 |
| 10/07/2015 | Coronation Road | Mangere Inlet, Mangere Bridge | -36.937349, 174.786838 | 0.25 | 9.37 | | 2.71 |
| 10/07/2015 | Peninsula Road | Pukaki Creek, Mangere | -36.982885, 174.799222 | 0.21 | 7.68 | | 1.98 |
| 10/07/2015 | Ihumatao Road | Ihumatao seashore | -36.990991, 174.743286 | 0.16 | 11.51 | | 2.06 |
| 10/07/2015 | Hanford Place | Puhinui Creek | -37.020841, 174.858409 | 0.22 | 9.16 | | 2.33 |
| 10/07/2015 | Sandwick Drive | Pahurehure Inlet, Manurewa | -37.040344, 174.875970 | 0.25 | 7.10 | | 2.04 |
| 7/09/2015 | Mary Place, Favona | Harania Creek, Mangere | -36.951673, 174.812821 | 0.19 | 10.58 | | 2.41 |
| 7/09/2015 | Dunsmuir Road | Taihiki River | -37.161984, 174.725717 | 0.32 | 9.43 | | 2.31 |
| 7/09/2015 | Te Toro point | Taihiki River, Harbour | -37.150691, 174.697052 | 0.23 | 5.90 | | 2.19 |
| 7/09/2015 | Mauku Bridge, Manukau | Taihiki River, upper catchment | -37.173125, 174.794499 | 0.17 | 8.87 | | 1.86 |
| **Mangawhai Harbour Estuary** | | | | | | | |
| 16/07/2015 | Cove Road | Tara Creek, (West) | -36.094944, 174.562831 | 0.22 | | 6.38 | 2.01 |
| 16/07/2015 | Jack Boyd | Tara Creek, (East) | -36.095966, 174.573002 | 0.18 | | 5.29 | 1.96 |
| 16/07/2015 | Molesworth Drive | Tara Creek, Molesworth Bridge (North) | -36.108047, 174.579077 | 0.20 | | 4.90 | 2.15 |
| 16/07/2015 | Molesworth Drive | Tara Creek, Molesworth Bridge (South) | -36.109912, 174.579699 | 0.16 | | 6.45 | 2.05 |
| 16/07/2015 | Pearson Street | Mangawhai Harbour | -36.120458, 174.575078 | 0.17 | | 5.32 | 1.98 |
| 16/07/2015 | Kedge Drive | Insley Channel (North arm) | -36.131841, 174.572365 | 0.18 | | 4.46 | 1.87 |
| 16/07/2015 | Insley Street | Insley Bridge | -36.131849, 174.579462 | 0.16 | | 5.07 | 1.83 |
| 16/07/2015 | Clarke Road | Insley Channel (South arm) | -36.137232, 174.576693 | 0.23 | | 2.95 | 1.81 |
| 16/07/2015 | Tern Point | Mangawhai Harbour | -36.117808, 174.586707 | 0.15 | | 3.78 | 1.84 |
| 16/07/2015 | Lincoln Street | Mangawhai Harbour | -36.107866, 174.596599 | 0.16 | | 7.01 | 2.10 |
| **Great Barrier Islands** | | | | | | | |
| 12/04/2015 | Motu Kaikoura Island | Port Fitzroy | -36.180500, 175.333370 | 0.14 | | 6.85 | 2.28 |
| 12/04/2015 | Great Barrier | Kiwiriki Bay | -36.206894, 175.354304 | 0.14 | | 6.64 | 2.05 |
| 12/04/2015 | Great Barrier | Kiwiriki Bay | -36.207946, 175.357988 | 0.17 | | 4.74 | 2.36 |
| 12/04/2015 | Great Barrier, old trees | Kiwiriki Bay Estuary | -36.208788, 175.358441 | 0.14 | | 4.12 | 2.17 |
| 12/04/2015 | Great Barrier, young trees | Kiwiriki Bay Estuary | -36.208798, 175.358624 | 0.14 | | 4.19 | 2.07 |

**Table 2: Historical Auckland Museum (AM) mangrove leaf total nitrogen (TN) and nitrogen stable isotope (δ^15^N) ratios .**

| **Catalogue number** | **Date collected** | **Location** | **TN, % dry**  **weight** | **δ^15^N, ‰ dry**  **weight** |
| --- | --- | --- | --- | --- |
| **Waitemata Harbour** | | | | |
| 11603 | 1863-1873 | NA | 2.54 | 9.01 |
| 264607 | 1885 | Waitemata Harbour | 2.69 | 9.12 |
| 132293 | 6/09/1942 | Takapuna,tidal estuary | 2.55 | 9.90 |
| 40247 | 3/09/1947 | Purewa Bush | 3.12 | 7.57 |
| 264609 | /06/1958 | Hobson Bay | 2.69 | 8.16 |
| 264610 | /06/1959 | Rosebank Creek, Avondale | 2.54 | 9.01 |
| 116494/5 | 16/06/1967 | Hobson Bay | 2.86 | 8.29 |
| 261846 | 10/03/1971 | Pollen Island | 1.99 | 7.56 |
| 130391 | 10/08/1972 | Hobson Bay, mudflats | 3.02 | 10.10 |
| 131931 | 8/05/1973 | Shore Road, Hobson bay | 2.37 | 5.19 |
| 217901 | 10/06/1973 | Pollen Island | 2.31 | 5.31 |
| 270916 | 29/04/1981 | Tuff Crater | 2.14 | 7.43 |
| 259324 | 18/03/1982 | Shore Road, Hobson Bay | 2.22 | 7.78 |
| 220693 | 26/03/1982 | Waitaramoa Reserve, Hobson Bay | 2.87 | 7.33 |
| 276242 | 3/05/1987 | Cox's Creek | 2.43 | 6.47 |
| 279002 | 15/04/1990 | Meola Creek | 2.27 | 8.25 |
| 31838 | NA | Rangitoto | 2.52 | 9.36 |
| 175670 | 11/05/1986 | north from White Beach, Rangitoto | 1.82 | 3.61 |
| 130398 | 21/08/1972 | Glenn Innes Domain | 2.61 | 7.59 |
| **Manukau Harbour** | | | | |
| 129240 | 16/11/1971 | Ihumatao street | 2.68 | 18.23 |
| 181001 | 4/02/1975 | Onehunga waterfront | 1.01 | 12.26 |
| 273891 | 17/07/1983 | Bottle Top Bay | 2.06 | 7.67 |
| **Great Barrier Island** | | | | |
| 130515 | 22/08/1972 | Whangaparapara (Inlet) | 2.64 | 7.41 |
